# P66 Adalimumab originator versus biosimilar in children and young people with JIA

**DOI:** 10.1093/rap/rkac067.066

**Published:** 2022-09-28

**Authors:** Lianne Kearsley-Fleet, Sarthak Jain, Eileen Baildam, Michael W Beresford, Sharon Douglas, Helen E Foster, Taunton R Southwood, Kimme L Hyrich

**Affiliations:** Centre for Epidemiology Versus Arthritis, The University of Manchester, Manchester, United Kingdom; Centre for Epidemiology Versus Arthritis, The University of Manchester, Manchester, United Kingdom; Department of Paediatric Rheumatology, Alder Hey Children's NHS Foundation Trust, Liverpool, United Kingdom; Department of Paediatric Rheumatology, Alder Hey Children's NHS Foundation Trust, Liverpool, United Kingdom; Institute of Life Course and Medical Specialities, University of Liverpool, Liverpool, United Kingdom; Scottish Network for Arthritis in Children (SNAC), Edinburgh, United Kingdom; Population and Health Institute, Newcastle University, Newcastle, United Kingdom; University of Birmingham, Birmingham, United Kingdom; Centre for Epidemiology Versus Arthritis, The University of Manchester, Manchester, United Kingdom; National Institute of Health Research Manchester Biomedical Research Centre, Manchester University NHS Foundation Trust, Manchester Academic Health Science Centre, Manchester, United Kingdom

## Abstract

**Introduction/Background:**

Biosimilar therapies are considered to have comparable efficacy to their originators and prescribing is encouraged in the UK for significant cost-savings to the NHS. However, real-world evidence comparing originators and biosimilars is limited, particularly in children and young people. The objective of this analysis was to compare the effectiveness of the anti-TNF adalimumab originator and a biosimilar in the treatment of JIA in children and young people, by comparing change in disease activity after six months.

**Description/Method:**

This analysis included children and young people with JIA from the Biologics for Children with Rheumatic Diseases (BCRD) study. Data are collected at the point of starting biologic therapy, and after 6 months, including patient demographics, biologic therapy, and disease activity. Patients were included if they were starting adalimumab (originator or biosimilar) as their first biologic. Patients with follow-up data at 6 months were assessed for outcomes at 6 months. Change in Juvenile Arthritis Disease Activity Score (JADAS-71) from baseline to 6 months was calculated and compared between therapies using linear regression. Multivariable logistical regression was used to compare remission (JADAS-71 ≤1) at 6 months between therapies. Both regression models were adjusted for baseline characteristics at the start of biologic therapy: age, gender, disease duration, ILAR category, history of uveitis, number of comorbidities (0/1/2+), and JADAS-71. Multiple imputation was used to account for missing data.

**Discussion/Results:**

A total of 457 patients were registered starting adalimumab as their first biologic: 413 on originator, 44 on biosimilar (Table). Of these, 63% were female, median age at start of therapy was 11 years old (IQR 6, 14), and median disease duration was 2 years (IQR 1, 5). The majority of patients had RF-negative polyarticular JIA (29%), persistent oligoarticular (20%) or extended oligoarticular JIA (18%). There were 47% of patients who had a history of uveitits when starting biologic therapy, and 68% reported at least one comorbidity. Baseline characters were similar between both therapies. There were 429 patients with follow-up data after six months of treatment: 393 on originator and 36 on biosimliar. The median JADAS-71 improved by -4.4 (IQR -9.9, -0.2) with no difference seen between the originator and the biosimilar patients (adjusted b-coefficient: -0.4; 95% CI -2.6, 1.8; p = 734). There were 36% of patients in remission, with no difference between the two therapies (odds ratio 1.2; 95% CI 0.5, 2.9; p = 0.543).

**Key learning points/Conclusion:**

There was no significant difference in disease activity response between children and young people with JIA treated with adalimumab originator versus biosimilar. These results support that the adalimumab biosimilar is similar in effectiveness to the originator in treating JIA, although more research is needed regarding safety and tolerability.

**P66 Table:** Baseline characteristics and 6-month outcomes

	**All patients**	**Originator**	**Biosimilar**
**Baseline Characteristics**			
**Patients**	457	413	44
**Gender**	290 (63%)	257 (62%)	33 (75%)
**Age**	11 (6, 14)	11 (6, 14)	11 (6, 13)
**Disease duration**	2 (1, 5)	2 (1, 5)	3 (1, 6)
	N = 446	N = 403	N = 43
**ILAR Category**			
Persistent Oligoarticular	20%	19%	27%
Extended Oligoarticular	18%	17%	20%
RF- Polyarticular	29%	29%	30%
RF+ Polyarticular	7%	7%	5%
Systemic	<1%	<1%	2%
Psoriatic	6%	6%	7%
Enthesitis-related	15%	16%	7%
Undifferentiated / missing	4%	5%	2%
**History of Uveitis**	47%	48%	35%
	N = 448	N = 405	N = 43
**Comorbidities**			
0	32%	31%	41%
1	44%	45%	41%
2+	24%	25%	18%
			
**6-month outcomes**			
**Patients**	429	393	36
**JADAS-71 (0-101)**			
Baseline, median (IQR)	7.8 (4.1, 14)	7.8 (4.0, 14)	8.3 (4.5, 11)
6 months, median (IQR)	2.5 (0.4, 5.6)	2.4 (0.4, 5.5)	3.7 (0.8, 5.7)
Change, median (IQR)	−4.4 (-9.9, -0.2)	−4.5 (-10, -0.2)	−3.9 (-6.9, -1.2)
Coef. (95% CI)	–	−1.8 (-5.5, 2.0)	Reference.
Adjusted* coef. (95% CI)	–	−0.4 (-2.6, 1.8)	Reference.
**JADAS-71 Remission**			
6 months, %	36%	36%	32%
Odds ratio (95% CI)	–	1.2 (0.5, 2.9)	Reference.
Adjusted* odds ratio (95% CI)	–	1.3 (0.5, 3.4)	Reference.

